# Precursors of interactional value formation through digital banking interactions

**DOI:** 10.12688/f1000research.161114.1

**Published:** 2025-02-26

**Authors:** Ramya Tauh, Savitha Basri

**Affiliations:** 1 Manipal School of Commerce and Economics, Manipal Academy of Higher Education, Manipal, Karnataka, 576104, India; 2 Manipal Academy of Banking, Manipal Academy of Higher Education, Bangalore, Karnataka, 560064, India

**Keywords:** Interactional value formation, banking, relationship quality, knowledge, communication, digital interactions

## Abstract

**Background:**

The dynamic nature of consumer behavior and the complex design of banking services make digital banking interactions challenging. Automated systems/responses lack a personal touch and may lead to miscommunication creating further difficulty in retaining customers. Hence, a co-creating interaction and interactional value formation (IVF) becomes a necessity for banks to have meaningful and experiential customer interactions. This article adds to the limited body of banking literature by explaining the precursors that influence IVF in digital banking interactions.

**Methods:**

442 digitally active banking customers of private, public, and small finance banks were selected from the Karnataka region to assess the relationship of precursors with IVF. A quantitative approach was adopted through a survey questionnaire to gather the responses. The data gathered was analyzed using SmartPLS 4.0 software.

**Findings:**

Knowledge has the most significant relationship with IVF. Customers perceive the seeking and sharing of information as the most critical for a co-creating interaction. Relationship quality moderately influences value formation in digital interactions; however, communication was not found to have a relationship with co-creation.

**Conclusions:**

The study will help bank managers and employees align their resources to develop knowledge and relationship aspects in their digital interactions to foster co-creation. Banks must move beyond frequent, generic updates or promotional messages that may overwhelm customers. Instead, they should adopt a more tailored approach, emphasizing relevance and value in their interactions to avoid perceptions of intrusion or inefficiency.

## Introduction

With the advancement of digitalization, banking interactions are also moving to digital space ( Ahmad et al., 2023). Even though banks have accelerated their digitization efforts across a range of services, from online account opening to payment collections, retaining customers in the digital age is another challenge. Effective customer experience management and exceptional service interactions form the cornerstone of the banking industry. A value co-creating interaction, characterized by the active participation of both parties, service providers and consumers ( Prahalad and Ramaswamy, 2004a, b; Ramaswamy and Ozcan, 2018; Alves, 2016; Vargo and Lusch, 2004; Grönroos, 1982, 2011; Vargo and Lusch, 2011; Payne et al., 2007), is a key to sustainable competitive advantage for banks. Consumers are no longer passive participants but are actively involved in creating value ( Karpen et al., 2011). This forms the basis of interactional value formation (IVF), which keeps dyadic interactions as its centerpiece ( Fan and Luo, 2020; Bonamigo et al., 2020; Breidbach and Maglio, 2016; Skålén et al., 2014; Plé and Chumpitaz Cáceres, 2010; Polo Peña et al., 2014; Echeverri and Skalen, 2011; Prahalad and Ramaswamy, 2004a, b; Gummesson and Mele, 2010; Lusch and Vargo, 2006; Echeverri and Skålén, 2011). Despite its significance, IVF is challenging in a complex service such as digital or virtual banking ( Li and Tuunanen, 2020). Consumers play a significant part in digital interactions alongside technology ( Agrawal and Rahman, 2015), and problems might occur when they lack the knowledge or ability to use customer support digital tools such as chatbots, video conversations, or emails. Another difficulty that consumers confront is digital illiteracy, which is defined as the inability to communicate service expectations and lack of the requisite abilities to navigate through internet channels ( Cetindamar Kozanoglu and Abedin, 2020; Kozanoglu et al., 2020; Tam et al., 2016; Pera et al., 2016; Plé, 2016).

The challenge of maintaining consumer engagement in the banking sector, traditionally reliant on human interaction ( Cook, 1987), is particularly pronounced in the digital era ( Brett, 2021). Although banks are trying to make these interactions easier by engaging in cocreating experiences for IVF, the reliability, promptness, simplicity, and convenience of these experiences vary significantly. Additionally, not all services ensure a uniform online experience across all offerings ( Northey et al., 2022; Mele, 2011). Consequently, ensuring consistency between online and offline channels becomes of paramount importance. Another challenge is posed by the changing financial behaviour of customers; they rely on technology for their basic financial needs and opt for physical interactions only for complicated solutions such as children’s education and retirement planning. Digital interactions are the new norm, and understanding their nuances for IVF is crucial. Therefore, this paper adopted an empirical lens to assess IVF in digital banking interactions. Previous literature provides empirical evidence from different industries ( Tabaeeian et al., 2022; Nangpiire et al., 2021; Schulz et al., 2021; Ogunbodede et al., 2021; Kirova, 2020; Pathak et al., 2020, 2021; Osei-Frimpong and Owusu-Frimpong, 2017; Merrilees et al., 2017; Mainardes et al., 2017; Makkonen and Olkkonen, 2017; Mayer et al., 2016; Nätti et al., 2014), such as healthcare ( Keeling et al., 2020; Beirão et al., 2017), professional financial services ( Chan et al., 2010) and tourism ( Echeverri and Skålén, 2021). However, we observed a gap in empirical studies that focus on banking interactions. Studies have included a single variable for testifying their impact on IVF, we have included several variables that are critical for digital banking interactions while attempting to provide a comprehensive view of IVF phenomenon.

Our paper analyses the influence of the identified precursors on IVF from the customer’s perspective. We adopted the Neghina et al. (2013) framework that highlights relationship quality, communication, and knowledge as the precursors. We attempted to answer the following questions:

a) Does relationship quality influence IVF?
b) Does communication affect IVF?
c) Does knowledge contribute to IVF?

Because most studies have employed samples of people from the West, there is a gap in our understanding of the Indian subcontinent. As research in IVF is in a nascent stage, we observed that banking and financial studies are few. More qualitative methods have been used by researchers to gather information, which could provide results that are incorrect or even deceptive. We intend to fill these gaps through this quantitative study that has taken Indian digital banking customers as its population. Co-creation considers customers as active contributors to value formation and the present study assists banks in revising the current practices necessary for a positive interactional experience by comprehending what factors contribute to IVF and effective interaction.

### Relationship quality

In dyadic interactions involving two or more parties, relationships or partnerships form the foundational base for value co-creation ( Ballantyne and Varey, 2006). This relationship dynamic is particularly significant in banking interactions, which involve sensitive and confidential information where trust, commitment, and connection are paramount ( Neghina et al., 2013). Ballantyne and Varey (2006) highlight the integral role of relationships, communication, and knowledge in Interactive Value Formation (IVF), while Mukherjee and Nath (2003) support this framework using trust-commitment theory. Trust, as a relationship variable, has been shown to co-create value across industries, as evidenced by Garanti (2023) in tourism interactions and Järvi et al. (2018) in qualitative studies exploring trust in co-creation processes. In banking, strong relationship quality reduces customer anxiety and fosters stability in interactions ( Morgan and Hunt, 1994). This is supported by Cambra-Fierro et al. (2019), who found that customers with relationship-oriented behavior enhance co-creation in online banking. Similarly, Osei-Frimpong et al. (2017) used the critical incident technique to validate the role of trust in enabling a safe environment for patient-doctor information sharing, which can be analogized to the banking sector’s trust-intensive interactions. Emotional connections between employees and customers also play a pivotal role in co-creation ( Salovey and Mayer, 1990), as observed in tourism and healthcare contexts ( Teng and Tsai, 2020; Tari Kasnakoglu, 2016).

Despite the increasing digitalization of banking services, physical interactions remain crucial, particularly for complex financial solutions, where customers tend to trust human advisors over automated or robotic alternatives ( Gremler and Gwinner, 2008). However, contradictory findings suggest that the relationship between trust and IVF is not universally positive. For instance, Gyllenhammar et al. (2023) observed an inverse relationship in public insurance services, where excessive trust may complicate processes like claim disbursement. Similarly, Mostafa and Ibrahim (2020), in an Egyptian banking context, reported no significant role of trust in customer co-creation.

These mixed findings underscore the complexity of relationship dynamics in IVF, suggesting that while trust and relationship quality are generally positive enablers of co-creation, their impact may vary based on contextual and cultural factors. Further research is needed to explore these nuances, particularly in digital banking, where trust mechanisms are evolving with technological advancements.

Therefore, we hypothesize that *H1: Relationship quality positively influences IVF.*

### Communication

Ballantyne and Varey (2006), Gustafsson et al. (2012), and Neghina et al. (2013) emphasize the critical role of communication in co-creation, proposing that bidirectional and transparent interaction fosters equal and meaningful participation from all parties. Empirical evidence from various industries substantiates the positive relationship between communication and Interactive Value Formation (IVF) ( Smith, 2013; Kashif and Zarkada, 2015; Malar et al., 2019; Yin et al., 2019; Echeverri and Salomonson, 2017; Chathoth et al., 2020; Keeling et al., 2020; Sthapit and Bjørk, 2020; Guan et al., 2022).

In digital banking, the absence of face-to-face interaction amplifies the importance of clear, bidirectional communication channels. The principles outlined by Ballantyne and Varey (2006), Gustafsson et al. (2012), and Neghina et al. (2013) are particularly relevant, as communication in this context must compensate for the lack of physical cues and traditional customer service interactions. Bidirectional communication ensures that both customers and banks engage in meaningful dialogue, fostering trust and co-creating value. In banking, communication gaps between employees and customers can significantly hinder value creation ( Kashif and Zarkada, 2015). Online banking requires dynamic and effective communication to facilitate co-creation ( Malar et al., 2019). Evidence from healthcare interactions underscores this need, as Keeling et al. (2020) highlight the positive impact of bidirectional communication on co-creation outcomes. Similarly, studies in the hospitality sector reveal the importance of communication, with Chathoth et al. (2020) employing quantitative methods and Boadi et al. (2020) and Sthapit and Bjørk (2020) using netnography approaches to confirm its role in enhancing customer co-creation.

Additional insights come from public transport services, where Echeverri and Salomonson (2017) and Guan et al. (2022) demonstrate a strong link between dialogue and IVF. These findings collectively highlight that effective communication is a universal driver of value co-creation across service industries, reinforcing its importance in the banking sector. Prioritizing interactive dialogue in digital banking contexts can bridge communication gaps and enhance the co-creation experience for customers and service providers alike.

Therefore, we hypothesize that *H2: Communication positively affects IVF.*

### Knowledge

Prahalad and Ramaswamy (2004a, b) and Neghina et al. (2013) identify three critical subconstructs of Interactive Value Formation (IVF): information sharing, information seeking ( Nonaka and Takeuchi, 1995), and feedback. Ballantyne and Varey (2006) further validated the significance of these dimensions through their conceptual framework. Despite this foundation, the literature predominantly focuses on employees’ perspectives regarding knowledge exchange (e.g., Chathoth et al., 2023; Guan et al., 2022; McNaughton et al., 2002), often underemphasizing the critical role of customer participation. Studies from diverse sectors underscore the positive relationship between customer engagement in knowledge-sharing activities and value co-creation ( Aarikka-Stenroos and Jaakkola, 2012; Agrawal and Rahman, 2015). For instance, Chaudhuri et al. (2023) employed quantitative methods to demonstrate the beneficial role of knowledge sharing in complex healthcare services. Similarly, in the post-service stage of the tourism industry, feedback emerges as a pivotal factor ( Codá et al., 2024).

Customer information-sharing traits also positively influence co-creation processes across various service domains. Wu et al. (2023) highlight this in alignment with findings by Järvi et al. (2018). Additionally, Guan et al. (2022) investigated the role of information symmetry, presenting evidence for its positive impact on IVF. However, this perspective has faced critique; Osei-Frimpong et al. (2015) observed that in healthcare, an elevated display of knowledge by patients could adversely affect interactions with physicians, leading to knowledge conflicts. Such conflicts underscore the nuanced and context-dependent nature of customer knowledge contributions to IVF. This complexity necessitates further exploration into how knowledge-sharing dynamics influence co-creation processes across different service contexts.

Based on the review, we hypothesize that *H3: Knowledge positively affects IVF.*

## Methods

### Ethical considerations

Before administering the survey questionnaire, written informed consent was obtained from all participants. The consent process began with an information sheet that outlined the research objectives, expected outcomes, and potential implications, followed by a section for participants to provide their informed consent. The form assured participants that all data collected would be used exclusively for research and publication purposes, with strict confidentiality maintained for personal information (see Extended Data, Ramya, 2025). Additionally, ethical approval for this empirical study in the Indian Banking industry was obtained from the Manipal Academy of Higher Education, India, Ethics Committee vide reference number MAHE/CDS/PHD/2021 dated 29 ^th^ Nov’21.

### Research design

A cross-sectional quantitative survey-based approach was adopted to collect data were collected over the period from 2022-2023 using a snowball sampling approach. The independent variables taken as “precursors” of IVF are relationship quality such as trust, connection and commitment, communication referring to direction, content and frequency, and knowledge that involves information sharing and feedback; the dependent variable is IVF.

### Measurement tools/scales

The questionnaire was prepared by adopting an established scale adapted from Neghina et al. (2013) on precursors such as relationship quality, communication, and knowledge. Yi and Gong’s (2013) scale was adapted for measuring co-creation in digital banking interactions. Different types of Likert scales were used (7-point and 5-point) to prevent response bias and obtain reliable results. The survey questionnaire can be found as *Extended data* ( Ramya, 2025).

To provide a holistic view of the customer’s perspective, we have included the digitally active customers of the private sector (ICICI Bank and Axis Bank), public sector (SBI and Bank of Baroda), and small finance banks (AU Small Finance Bank) of the Indian banking system. We visited the regional offices of the selected banks and requested the management to use us as an opportunity to obtain customer’s perceptions of digital interactions. Post approval, we met the customers in the respective branches who were digitally active in bank interactions. The study considered interactions through mail, phone, chatbots, WhatsApp, and text messages as digital interactions. To ensure anonymity, a pilot survey was conducted to refine the process and identify potential challenges for the final study. The pilot sample comprised 10% of the final dataset of 442 participants and was selected using the snowball sampling technique. The initial list of participants was asked to provide three customer references, who were then contacted via email to obtain consent for participation. Those who agreed were further asked to share three additional references, enabling the collection of the final sample after applying the necessary filtration criteria. To minimize response bias in the final survey, we employed concise questions, reverse-coded items, and avoided leading language in the questionnaire.

### Sample size

The sample size consisted of 442 digitally active bank customers of private, public, and small finance banks in the Karnataka region. Survey questionnaires were shared online with the bank customers.

### Data analysis

PLS-SEM 4.0 was employed to assess the measurement and structural model ( Ringle et al., 2024). The copyright license for the SmartPLS software was bought under order ID 2024-06155 dated 19 ^th^ Aug’24. The measurement model establishes the reliability and validity of the constructs, and the structural model determines the significance of the hypothesized relationships ( Hair et al., 2013; Henseler et al., 2012). Alternatively, JASP, an open-source software, can be used to conduct Structural Equation Modeling (SEM) through the Iavaan R package, which follows a covariance-based approach.

## Results

The validity and reliability of the model of measurement model are estimated based on:

### Internal consistency reliability

A measurement model is said to have acceptable internal consistency reliability when the composite reliability (CR) is higher than 0.7. The CR of each of the indicators ranges between 0.888 and 0.958, which is higher than 0.7. Cronbach’s alpha values are also within the acceptable range, higher than 0.7 ( Henson, 2001), for all the constructs. AVE values were higher than 0.5; hence, convergent validity was established (refer to Table 1).

**Table 1.** Construct reliability and convergent validity.

	Composite reliability (rho_c)	Cronbach's alpha	Average variance extracted (AVE)
Relationship Quality	0.965	0.956	0.822
Communication	0.958	0.950	0.741
Knowledge	0.918	0.888	0.690

*Outer loadings*

Outer loadings reflect the absolute contribution of the indicators to the latent variables ( Hair, 2013). Based on the outer loadings, RQT1, RQT2, RQT3, RQC1, RQC2, and RQC3 in RQ, C3, C4, C5, C6, C8, C9, C10, and C11 in C, and K2, K3, K4, K5, and K6 in K were retained. RQT1, RQT2, RQT3, RQC1, RQC2, and RQC3 values were higher than 0.7, ranging from 0.777 to 0.963. The indicators of the construct ‘communication’ were also found to contribute to the latent variable, as the outer loadings ranged from 0.799 to 0.929. The ‘knowledge’ indicators were also within an acceptable range, ranging from 0.814 to 0.862. All sub-constructs of IVF except IVFT reported outer loading of less than 0.7, which is 0.669 (Refer to Table 2).

**Table 2.** Construct validity of survey items.

	RQ	C	K	IVF
RQC1	0.938			
RQC2	0.777			
RQC3	0.920			
RQT1	0.895			
RQT2	0.963			
RQT3	0.935			
C3		0.877		
C4		0.799		
C5		0.919		
C6		0.807		
C8		0.880		
C9		0.817		
C10		0.929		
C11		0.850		
K2			0.862	
K3			0.815	
K4			0.837	
K5			0.825	
K6			0.814	
IVFA				0.861
IVFPI				0.821
IVFRB				0.826
IVFT				0.669

*Construct validity*

To validate the discriminant validity, tests, namely, average variance extracted (AVE) and HTMT, were observed, and multicollinearity was checked (VIF). To assess the discriminant validity, HTMT values (acceptable range >0.85) were observed. HTMT (heterotrait-monitrait ratio) measures the similarity observed between the latent variables. HTMT values were reported to be lower than 0.90 ( Hair et al., 2013); hence, discriminant validity was established between the proposed constructs, RQ, C, and K (Refer to Table 3).

**Table 3.** Discriminant validity.

	Heterotrait-monotrait ratio (HTMT)
RQ <-> K	0.776
RQ <-> C	0.798
K <-> C	0.872

The next test was VIF (variance inflation factor). It assesses the multicollinearity between the indicators. VIF values of 5 or higher indicate possible issues with collinearity problems. The VIF values obtained were below 5, except for RQT2 (9.734), RQT3 (5.922), C5 (5.859) and C10 (6.256), whose VIF values were higher than 5 (refer to Table 4).

**Table 4.** Multi-collinearity of constructs.

VIF - Inner Model	
	IVF
RQ	2.551
C	3.475
K	3.023

IVF was considered a second-order construct, and all four latent variables (IVFA, IVFPI, IVFRB, IVFT) were taken by comprising their respective indicators for the two-stage disjoint approach. It is a reflective-formative construct; hence, outer weights for latent scores were checked. Since all sub-constructs had outer weights lower than 0.5, bootstrapping was carried out and outer loadings were checked. The loadings were found to be above 0.5 and significant. Hence, these sub-constructs were considered in the higher-order construct IVF.

*Model fit*

Model fit was tested to check the goodness of fit measure of the proposed model. We tested SRMR (<0.08), NFI (>.90), R square (0.75=substantial, 0.50=moderate, 0.25=weak) ( Hair et al., 2013), and f square (>=0.02=small, >=0.15=medium, >=0.35=large). The SRMR value was 0.066, and the NFI was 0.872, which validates the goodness of fit. The R ^2^ value of 0.549 indicates that the independent variables (RQ, C, and K) moderately impact IVF. Another test conducted was f-square. It explains the impact of latent variables. The values reveal that relationship quality and knowledge have a medium effect on the model, whereas communication has a small effect (Refer to Table 5).

**Table 5.** Effect sizes (f ^2^) of relationship quality, communication and knowledge on IVF.

Effect size f ^2^	F-square
RQ -> IVF	0.104
C -> IVF	0.001
K -> IVF	0.132

Finally, path analysis was examined. It is used to explain the relationship between the constructs. We conducted bootstrapping to test the statistical significance of the path coefficients. Knowledge (K) was found to have a more significant influence on IVF than RQ, with the path coefficient of the former (0.424) being higher than that of the latter (0.345). In the current study, RQ➔IVF (p=0.000, t=7.073, path coefficient=0.345), Communication➔IVF (p=0.659, t=0.441, path coefficient=0.034) and K➔IVF (p=0.000, t=6.850, path coefficient=0.424) values signify that H1 and H3 are accepted, as RQ and K significantly and positively affect IVF, and H2 is rejected, as communication does not affect IVF.

## Discussion

The purpose of the study was to develop a model for customer value formation in digital banking interactions. Building on S-D logic, social exchange theory, and value co-creation literature, we proposed key antecedents within the context of digital banking interactions, that shape the outcomes of these interactions. We analyzed the empirical data of 442 digitally active customers in the Karnataka region using structural equation modeling (SEM) and confirmed the hypothesized relationships.

The study identifies relationship quality, communication, and knowledge as pivotal precursors influencing IVF outcomes in digital banking. Although the model is novel in digital banking, the results are in line with existing literature in many ways. Our findings reveal that customers perceive knowledge as the most critical antecedent to IVF. This finding reflects the fundamental expectation of digital customers from bank employees that they possess and effectively disseminate relevant financial expertise, enabling customers to address their financial concerns. Customers actively engage in value co-creation by seeking solutions to financial challenges, sharing requisite information during online interactions, such as through chatbot interfaces, and exploring self-service digital resources. The findings are consistent with extant literature (e.g., Järvi et al., 2018; Chathoth et al., 2020; Borba et al., 2021; Guan et al., 2022; Wu et al., 2023), which highlights the integral role of information exchange and solution-sharing in facilitating value co-creation. However, the study also acknowledges the challenges inherent to digital banking, such as information asymmetry, withholding of critical information, and potential miscommunication introduced by technological interfaces.

This research contributes to the literature by underscoring the primacy of knowledge in digital banking IVF and highlighting the need for enhanced communication strategies and relational quality to optimize co-creative outcomes. The implications are particularly relevant given the regulatory and technological complexities that characterize contemporary banking environments.

Studies have found relationship quality influences co-creation ( Teng and Tsai, 2020; Tarı Kasnakoğlu & Mercan, 2020; Tari Kasnakoglu, 2016). Our results are in line with the existing literature demonstrating that a digital banking interaction involving confidential information demands connection and the existence of a relationship between bank employees and customers. Positive feelings such as trust and happiness build co-creating interactions in digital exchanges such as social media platforms ( Frau et al., 2023). We attempted to answer the research question on the relationship between RQ and IVF, for which we employed aspects such as trust and connection. The study confirms that RQ positively and significantly impacts IVF, like the results obtained from the previous literature. Bank customers value trust and connection while interacting with bank employees on digital platforms. The literature has employed social exchange theory to corroborate the importance of relationship-seeking behaviors in co-creating value ( Cambra-Fierro et al., 2019). Our results are in line with other studies ( Mukherjee and Nath, 2003; Järvi et al., 2018; Osei-Frimpong et al., 2015; Gremler and Gwinner, 2008; and Chathoth et al., 2020) that place trust and connection in digital banking as the key antecedents.

The current study found no significant relationship between the construct ofcommunication and Interactive Value Formation (IVF). This outcome may be explained by two key factors. First, customers appear to regard bidirectional communication with their banks as a fundamental aspect of service delivery rather than as a contributor to value creation. Such interactions are primarily functional, aimed at addressing financial needs, and may not be perceived as adding intrinsic value. Second, frequent communications, including digital messages or updates, can be perceived negatively. Customers might interpret these interactions as indicators of inefficiency in resolving issues or as unwarranted intrusions into their personal space. The overabundance of promotional content or service-related updates can further amplify this perception, rendering communication burdensome rather than beneficial. As a result, the findings suggest thatcommunication is not regarded as a pivotal antecedent to value formation in the context of online customer-bank interactions.

The current study provides a guide to banks on the factors that can lead to co-creating interactions. Customers place knowledge as the most impacting variable when interacting with bank employees digitally. The bank management must consider information-seeking and processing behaviors as key skills for the employees in customer interaction roles. Knowledge in banking can soon be outdated as the regulations keep on changing. Bank managers and organizations to focus more on the training aspect that would help bank employees enhance their knowledge and be informed on the latest changes in the banking industry. Customers explore online bank channels such as websites, blogs, and videos to seek solutions to their issues, organizations must strive to make these journeys smoother by upgrading their platforms.

Another finding from our study states that relationship quality positively and significantly affects IVF. Knowing that the customers value trust and connection in a digital banking interaction, bank organizations can sensitize the employees to imbibe these behaviours while interacting with the customers. While serving the customers, bank employees should have the right intention to serve and must consider the customer’s interest. Methods of strengthening trust and connection between banks and customers should be developed by the banking organizations.

### Limitations and future scope

Although the study incorporates a diverse set of banks, the sample is limited to Indian banks, which may restrict the generalizability of the findings to other geographical contexts. This study utilizes cross-sectional data, which precludes drawing causal inferences between predictor and criterion variables. The study considers the perceptual model that relies on self-reported measures. Another limitation could be that the responses might be influenced by individual biases and social desirability. Future research could expand the scope to validate the proposed framework in different regions, enhancing its applicability across varied banking environments. Furthermore, while this study centers on digital interactions, future investigations could incorporate in-person banking interactions to provide a more comprehensive understanding of Interactive Value Formation (IVF). The present framework examines precursors to IVF, yet there are likely additional factors, such as situational variables and cultural orientations, that could influence this relationship. Future studies are encouraged to explore the moderating and mediating effects of such variables, thereby offering a deeper understanding of the dynamics underlying value formation.

## Conclusion

This study contributes to the growing body of literature on Interactive Value Formation (IVF) by presenting empirical evidence from the digital banking industry. The rapid digitalization of banking services has introduced challenges, including understanding customer needs, fostering rapport, and facilitating effective information exchange in technology-mediated interactions. These complexities underscore the necessity of examining key antecedents of IVF within digital banking contexts. The findings of the study reveal that knowledge is a critical determinant, significantly and positively influencing IVF. This highlights the importance of equipping customers with relevant financial insights and empowering them to engage in co-creative processes. Relationship quality was found to have a moderate yet positive impact on IVF, emphasizing the need to cultivate meaningful relationships in virtual environments. In contrast, communication did not exhibit a statistically significant relationship with IVF. The result challenges traditional frameworks prioritizing communication in service interactions and invites further investigation into its role in digitalized contexts.

The study’s implications are both theoretical and practical. Theoretically, it expands the IVF framework by integrating evidence from the banking sector, a domain shaped by stringent regulations and advanced technological interfaces. Practically, the findings provide actionable insights for financial institutions to enhance customer experiences and optimize the performance of digital banking systems. Banks can better meet customer expectations and facilitate effective value co-creation by prioritizing knowledge dissemination and fostering trust and connection. This research offers a foundation for future studies to explore additional antecedents and contextual variables influencing IVF in digital settings, further enriching our understanding of value formation in technologically mediated service ecosystems.

## Ethics and consent

Ethical approval for this empirical study in the Indian Banking industry was obtained from the Manipal Academy of Higher Education, India, Ethics Committee vide reference number MAHE/CDS/PHD/2021 dated 29 ^th^ Nov’21. Written informed consent was obtained from all participants

## Author contributions

Conceptualization, Ramya Tauh, Savitha Basri.

Methodology, Data Analysis and Interpretation, Ramya Tauh, Savitha Basri.

Writing- Original Draft Preparation, Ramya Tauh, Savitha Basri.

Writing – Review & Editing, Ramya Tauh, Savitha Basri.

## Data Availability

Mendeley Data: Raw data CSV processed for running through SmartPLS -“Precursors of Interactional Value Formation through Digital Banking Interactions”. https://doi.org/10.17632/ytjm37534f.1 ( Ramya & Basri, 2025a). Mendeley Data: Customer survey questionnaire - “Precursors of Interactional Value Formation through Digital Banking Interactions”. https://doi.org/10.17632/w693mpfcbm.3 ( Ramya, 2025). Mendeley Data: Participation Information Sheet and Informed Consent Form - “Precursors of Interactional Value Formation through Digital Banking Interactions”. https://doi.org/10.17632/dn473fjw79.2 ( Ramya & Basri, 2025b). Data are available under the terms of the Creative Commons Attribution 4.0 International license (CC-BY 4.0).

## References

[ref2] Aarikka-Stenroos L Jaakkola E : Value co-creation in knowledge intensive business services: A dyadic perspective on the joint problem solving process. *Ind. Mark. Manag.* 2012;41(1):15–26. 10.1016/j.indmarman.2011.11.008

[ref3] Agrawal AK Rahman Z : Roles and Resource Contributions of Customers in Value Co-creation. *Int. Strat. Manag. Rev.* 2015;3(1-2):144–160. 10.1016/j.ism.2015.03.001

[ref4] Ahmad R Nawaz MR Ishaq MI : Social exchange theory: Systematic review and future directions. *Front. Psychol.* 2023;13. 10.3389/fpsyg.2022.1015921 36710813 PMC9878386

[ref5] Alves H Fernandes C Raposo M : Value co-creation: Concept and contexts of application and study. *J. Bus. Res.* 2016;69(5):1626–1633. 10.1016/j.jbusres.2015.10.029

[ref6] Ballantyne D Varey RJ : Creating value-in-use through marketing interaction: the exchange logic of relating, communicating and knowing. *Mark. Theory.* 2006;6(3):335–348. 10.1177/1470593106066795

[ref7] Beirão G Patrício L Fisk RP : Value co-creation in service ecosystems. *J. Serv. Manag.* 2017;28(2):227–249. 10.1108/josm-11-2015-0357

[ref9] Boadi EA He Z Boadi EK : Customer value co-creation and employee silence: Emotional intelligence as explanatory mechanism. *Int. J. Hosp. Manag.* 2020;91:102646. 10.1016/j.ijhm.2020.102646

[ref10] Bonamigo A Dettmann B Frech CG : Facilitators and inhibitors of value co-creation in the industrial services environment. *J. Serv. Theory Pract.* 2020;30(6):609–642. 10.1108/jstp-03-2020-0061

[ref78] Borba M Borba M Engelbrecht J Ciscar S : Using Digital Technology and Blending to Change the Mathematics Classroom and Mathematics Teacher Education. 2021. 10.1007/978-3-030-80230-1_2

[ref79] Brett H : Digital Banking as The New Normal in 2021: What to Expect from Banks. *Forbes.* 2021. Reference Source

[ref11] Breidbach CF Maglio PP : Technology-enabled value co-creation: An empirical analysis of actors, resources, and practices. *Ind. Mark. Manag.* 2016;56:73–85. 10.1016/j.indmarman.2016.03.011

[ref12] Cambra-Fierro J Xuehui Gao L Melero-Polo I : What drives consumers’ active participation in the online channel? Customer equity, experience quality, and relationship proneness. *Electron. Commer. Res. Appl.* 2019;35:100855. 10.1016/j.elerap.2019.100855

[ref13] Cetindamar Kozanoglu D Abedin B : Understanding the role of employees in digital transformation: conceptualization of digital literacy of employees as a multi-dimensional organizational affordance. *J. Enterp. Inf. Manag.* 2020;34:1649–1672. 10.1108/jeim-01-2020-0010

[ref14] Chan KW Yim CK(B) Lam SSK : Is Customer Participation in Value Creation a Double-Edged Sword? Evidence from Professional Financial Services Across Cultures. *J. Mark.* 2010;74(3):48–64. 10.1509/jmkg.74.3.48

[ref80] Chathoth PK Ungson GR : Service agents’ risk behavior and implications for co-creation. *Int. J. Contemp. Hosp. Manag.* 2023;35(9):3344–3359. 10.1108/IJCHM-06-2022-0730

[ref15] Chathoth PK Harrington RJ Chan ESW : Situational and personal factors influencing hospitality employee engagement in value co-creation. *Int. J. Hosp. Manag.* 2020;91:102687. 10.1016/j.ijhm.2020.102687

[ref16] Chaudhuri A Naseraldin H Narayanamurthy G : Healthcare 3D printing service innovation: Resources and capabilities for value Co-creation. *Technovation.* 2023;121:102596. 10.1016/j.technovation.2022.102596

[ref81] Codá RC Silva Farias J Dias C : Interactive value formation and lessons learned from Covid-19: The Brazilian case. *J. Qual. Assur. Hosp. Tour.* 2024;25(3):487–513. 10.1080/1528008X.2022.2135057

[ref17] Cook KS : *Social Exchange Theory.* SAGE Publications;1987. Incorporated.

[ref18] Echeverri P Salomonson N : Bi-directional and stratified demeanour in value forming service encounter interactions. *J. Retail. Consum. Serv.* 2017;36:93–102. 10.1016/j.jretconser.2017.01.007

[ref19] Echeverri P Skålén P : Co-creation and co-destruction: A practice-theory based study of interactive value formation. *Mark. Theory.* 2011;11(3):351–373. 10.1177/1470593111408181

[ref20] Echeverri P Skålén P : Value co-destruction: Review and conceptualization of interactive value formation. *Mark. Theory.* 2021;21:227–249. 10.1177/1470593120983390

[ref21] Fan X Luo Y : Value Co-Creation: A Literature Review. *Open J. Soc. Sci.* 2020;08(02):89–98. 10.4236/jss.2020.82008

[ref22] Frau M Cabiddu F Frigau L : How emotions impact the interactive value formation process during problematic social media interactions. *J. Res. Interact. Mark.* 2023;17:773–793. 10.1108/jrim-06-2022-0186

[ref82] Garanti Z : Value co-creation in smart tourism destinations. *Worldwide Hospitality and Tourism Themes.* 2023;15(5):468–475. 10.1108/WHATT-06-2023-0070

[ref23] Gremler DD Gwinner KP : Rapport-Building Behaviors Used by Retail Employees. *J. Retail.* 2008;84(3):308–324. 10.1016/j.jretai.2008.07.001

[ref24] Grönroos C : An Applied Service Marketing Theory. *Eur. J. Mark.* 1982;16(7):30–41. 10.1108/eum0000000004859

[ref25] Grönroos C : Value co-creation in service logic: A critical analysis. *Mark. Theory.* 2011;11(3):279–301. 10.1177/1470593111408177

[ref26] Guan X Gong J Huan T-C : Trick or treat! How to reduce co-destruction behavior in tourism workplace based on conservation of resources theory? *J. Hosp. Tour. Manag.* 2022;52:42–49. 10.1016/j.jhtm.2022.05.016

[ref27] Gummesson E Mele C : Marketing as Value Co-creation Through Network Interaction and Resource Integration. *J. Bus. Mark. Manag.* 2010;4(4):181–198. 10.1007/s12087-010-0044-2

[ref28] Gustafsson A Kristensson P Witell L : Customer co-creation in service innovation: a matter of communication? *J. Serv. Manag.* 2012;23(3):311–327. 10.1108/09564231211248426

[ref83] Gyllenhammar D Eriksson E Löfgren M : Value creation and destruction involving multiple public service organizations: a focus on frontline employees. *Public Manag. Rev.* 2023:1–22. 10.1080/14719037.2023.2206398

[ref84] Hair J Ringle C Sarstedt M : Partial Least Squares Structural Equation Modeling: Rigorous Applications, Better Results and Higher Acceptance. *Long Range Plann.* 2013;46:1–12. 10.1016/j.lrp.2013.08.016

[ref85] Henseler J Ringle C Sarstedt M : *Using Partial Least Squares Path Modeling in International Advertising.* Research: Basic Concepts and Recent Issues 2012. 10.4337/9781848448582.00023

[ref86] Henson RK : Understanding Internal Consistency Reliability Estimates: A Conceptual Primer on Coefficient Alpha. *Meas. Eval. Couns. Dev.* 2001;34(3):177–189. 10.1080/07481756.2002.12069034

[ref29] Järvi H Kähkönen A-K Torvinen H : When value co-creation fails: Reasons that lead to value co-destruction. *Scand. J. Manag.* 2018;34(1):63–77. 10.1016/j.scaman.2018.01.002

[ref30] Karpen IO Bove LL Lukas BA : Linking Service-Dominant Logic and Strategic Business Practice. *J. Serv. Res.* 2011;15(1):21–38. 10.1177/1094670511425697

[ref31] Kashif M Zarkada A : Value co-destruction between customers and frontline employees. *Int. J. Bank Mark.* 2015;33(6):672–691. 10.1108/ijbm-09-2014-0121

[ref33] Keeling DI Keeling K Ruyter K de : How value co-creation and co-destruction unfolds: a longitudinal perspective on dialogic engagement in health services interactions. *J. Acad. Mark. Sci.* 2020;49(2):236–257. 10.1007/s11747-020-00737-z

[ref34] Kirova V : Value co-creation and value co-destruction through interactive technology in tourism: the case of “La Cité du Vin” wine museum, Bordeaux, France. *Curr. Issue Tour.* 2020;1–14. 10.1080/13683500.2020.1732883

[ref35] Lehtinen U Lehtinen JR : Two Approaches to Service Quality Dimensions. *Serv. Ind. J.* 1991;11(3):287–303. 10.1080/02642069100000047

[ref36] Li M Tuunanen T : Actors’ Dynamic Value Co-creation and Co-destruction Behavior in Service Systems: A Structured Literature Review. *Proceedings of the Annual Hawaii International Conference on System Sciences.* 2020. 10.24251/hicss.2020.143

[ref37] Lusch RF Vargo SL : Service-dominant logic: reactions, reflections and refinements. *Mark. Theory.* 2006;6(3):281–288. 10.1177/1470593106066781

[ref38] Smith AM : The value co-destruction process: a customer resource perspective. *Eur. J. Mark.* 2013;47(11/12):1889–1909. 10.1108/ejm-08-2011-0420

[ref39] Mainardes EW Teixeira A Romano PCdS : Determinants of co-creation in banking services. *Int. J. Bank Mark.* 2017;35(2):187–204. 10.1108/ijbm-10-2015-0165

[ref40] Makkonen H Olkkonen R : Interactive value formation in interorganizational relationships. *Mark. Theory.* 2017;17(4):517–535. 10.1177/1470593117699661

[ref41] Malar DA Arvidsson V Holmstrom J : Digital Transformation in Banking: Exploring Value Co-Creation in Online Banking Services in India. *J. Glob. Inf. Technol. Manag.* 2019;22(1):7–24. 10.1080/1097198x.2019.1567216

[ref42] Mayer JD Caruso DR Salovey P : The Ability Model of Emotional Intelligence: Principles and Updates. *Emot. Rev.* 2016;8(4):290–300. 10.1177/1754073916639667

[ref43] McNaughton RB Osborne P Imrie BC : Market-oriented value creation in service firms. *Eur. J. Mark.* 2002;36(9/10):990–1002. 10.1108/03090560210437299

[ref44] Mele C : Conflicts and value co-creation in project networks. *Ind. Mark. Manag.* 2011;40(8):1377–1385. 10.1016/j.indmarman.2011.06.033

[ref45] Merrilees B Miller D Yakimova R : The role of staff engagement in facilitating staff-led value co-creation. *J. Serv. Manag.* 2017;28(2):250–264. 10.1108/josm-10-2015-0326

[ref47] Morgan RM Hunt SD : The Commitment-Trust Theory of Relationship Marketing. *J. Mark.* 1994;58(3):20–38. 10.1177/002224299405800302

[ref48] Mostafa RHA Ibrahim MM : The effects of customer equity and religious motivation on customer retention and switching intention. *J. Islam. Mark.* 2020;11(6):1873–1891. 10.1108/jima-06-2019-0136

[ref89] Mukherjee A Nath P : A model of trust in online relationship banking. *Int. J. Bank Mark.* 2003;21(1):5–15. 10.1108/02652320310457767

[ref49] Nangpiire C Silva J Alves H : Customer engagement and value co-creation/destruction: the internal fostering and hindering factors and actors in the tourist/hotel experience. *J. Res. Interact. Mark.* 2021;16:173–188. 10.1108/jrim-05-2020-0104

[ref50] Nätti S Pekkarinen S Hartikka A : The intermediator role in value co-creation within a triadic business service relationship. *Ind. Mark. Manag.* 2014;43(6):977–984. 10.1016/j.indmarman.2014.05.010

[ref51] Neghina C Caniëls MCJ Bloemer JMM : Value co-creation in service interactions. *Mark. Theory.* 2013;15(2):221–242. 10.1177/1470593114552580

[ref52] Nonaka I Takeuchi H : *The Knowledge-Creating Company.* Oxford University Press;1995.

[ref53] Northey G Hunter V Mulcahy R : Man vs machine: how artificial intelligence in banking influences consumer belief in financial advice. *Int. J. Bank Mark.* 2022;40:1182–1199. 10.1108/ijbm-09-2021-0439

[ref54] Ogunbodede O Papagiannidis S Alamanos E : Value co-creation and co-destruction behaviour: Relationship with basic human values and personality traits. *Int. J. Consum. Stud.* 2021. 10.1111/ijcs.12757

[ref55] Osei-Frimpong K Owusu-Frimpong N : Value Co-Creation in Health Care: A Phenomenological Examination of the Doctor-Patient Encounter. *J. Nonprofit Publ. Sect. Market.* 2017;29(4):365–384. 10.1080/10495142.2017.1326356

[ref90] Osei-Frimpong K Wilson A Owusu-Frimpong N : Service experiences and dyadic value co-creation in healthcare service delivery: a CIT approach. *Journal of Service Theory and Practice.* 2015;25(4):443–462. 10.1108/JSTP-03-2014-0062

[ref56] Pathak B Ashok M Leng Tan Y : Value co-creation in the B2B context: a conceptual framework and its implications. *Serv. Ind. J.* 2021;42:178–205. 10.1080/02642069.2021.1989414

[ref57] Pathak B Ashok M Tan YL : Value co-destruction: Exploring the role of actors’ opportunism in the B2B context. *Int. J. Inf. Manag.* 2020;52:102093. 10.1016/j.ijinfomgt.2020.102093

[ref58] Payne AF Storbacka K Frow P : Managing the co-creation of value. *J. Acad. Mark. Sci.* 2007;36(1):83–96. 10.1007/s11747-007-0070-0

[ref59] Pera R Occhiocupo N Clarke J : Motives and resources for value co-creation in a multi-stakeholder ecosystem: A managerial perspective. *J. Bus. Res.* 2016;69(10):4033–4041. 10.1016/j.jbusres.2016.03.047

[ref60] Plé L : Studying customers’ resource integration by service employees in interactional value co-creation. *J. Serv. Mark.* 2016;30(2):152–164. 10.1108/jsm-02-2015-0065

[ref61] Plé L Chumpitaz Cáceres R : Not always co-creation: introducing interactional co-destruction of value in service-dominant logic. *J. Serv. Mark.* 2010;24(6):430–437. 10.1108/08876041011072546

[ref62] Polo Peña AI Frías Jamilena DM Rodríguez Molina MÁ : Value co-creation via information and communications technology. *Serv. Ind. J.* 2014;34(13):1043–1059. 10.1080/02642069.2014.939641

[ref63] Prahalad CK Ramaswamy V : Co-creation experiences: The next practice in value creation. *J. Interact. Mark.* 2004a;18(3):5–14. 10.1002/dir.20015

[ref64] Prahalad CK Ramaswamy V : Co-creating Unique Value with Customers. *Strateg. Leadersh.* 2004b;32(3):4–9. 10.1108/10878570410699249

[ref66] Ramaswamy V Ozcan K : What is co-creation? An interactional creation framework and its implications for value creation. *J. Bus. Res.* 2018;84:196–205. 10.1016/j.jbusres.2017.11.027

[ref91] Ramya R Basri S : Precursors of Interactional Value Formation through Digital Banking Interactions. *Mendeley Data* 2025a;V1 10.17632/ytjm37534f.1 PMC12163366 40521390

[ref92] Ramya R Basri S : Precursors of Interactional Value Formation through Digital Banking Interactions. *Mendeley Data* 2025b;V2 10.17632/dn473fjw79.2 PMC12163366 40521390

[ref93] Ramya R : Precursors of Interactional Value Formation through Digital Banking Interactions. *Mendeley Data* 2025;V3 10.17632/w693mpfcbm.3 PMC12163366 40521390

[ref94] Ringle CM Wende S Becker JM : SmartPLS 4. SmartPLS;2024. Reference Source

[ref67] Salovey P Mayer JD : Emotional intelligence. *Imagin. Cogn. Pers.* 1990;9(3):185–211. 10.2190/DUGG-P24E-52WK-6CDG

[ref68] Schulz T Zimmermann S Böhm M : Value co-creation and co-destruction in service ecosystems: The case of the Reach Now app. *Technol. Forecast. Soc. Chang.* 2021;170:120926. 10.1016/j.techfore.2021.120926

[ref69] Skålén P Gummerus J Koskull C von : Exploring value propositions and service innovation: a service-dominant logic study. *J. Acad. Mark. Sci.* 2014;43(2):137–158. 10.1007/s11747-013-0365-2

[ref70] Sthapit E Bjørk P : Interactive value formation: drivers and outcomes from Airbnb guests’ perspectives. *Scand. J. Hosp. Tour.* 2020;21:129–147. 10.1080/15022250.2020.1828163

[ref71] Tabaeeian RA Yazdi A Mokhtari N : Host-tourist interaction, revisit intention and memorable tourism experience through relationship quality and perceived service quality in ecotourism. *J. Ecotour.* 2022;22:406–429. 10.1080/14724049.2022.2046759

[ref72] Tam JLM Sharma P Kim N : Attribution of success and failure in intercultural service encounters: the moderating role of personal cultural orientations. *J. Serv. Mark.* 2016;30(6):643–658. 10.1108/jsm-01-2015-0010

[ref73] Teng H-Y Tsai C-H : Can tour leader likability enhance tourist value co-creation behaviors? The role of attachment. *J. Hosp. Tour. Manag.* 2020;45:285–294. 10.1016/j.jhtm.2020.08.018

[ref88] Tari Kasnakoglu B : Antecedents and consequences of co-creation in credence-based service contexts. *Serv. Ind. J.* 2016;36(1–2):1–20. 10.1080/02642069.2016.1138472

[ref87] Tarı Kasnakoğlu B Mercan H : Co-creating positive outcomes in higher education: are students ready for co-creation? *J. Mark. High. Educ.* 2020;32(1):73–88. 10.1080/08841241.2020.1825031

[ref74] Vargo SL Lusch RF : Evolving to a New Dominant Logic for Marketing. *J. Mark.* 2004;68(1):1–17. 10.1509/jmkg.68.1.1.24036

[ref75] Vargo SL Lusch RF : It’s all B2B … and beyond: Toward a systems perspective of the market. *Ind. Mark. Manag.* 2011;40(2):181–187. 10.1016/j.indmarman.2010.06.026

[ref76] Wu C Jiang S Zhou Y : Consumer engagement behavior in the value co-creation process of healthcare services: a scoping review. *ASLIB Proc.* 2023. 10.1108/ajim-10-2022-0443

[ref77] Yi Y Gong T : Customer value co-creation behavior: Scale development and validation. *J. Bus. Res.* 2013;66(9):1279–1284. 10.1016/j.jbusres.2012.02.026

[ref95] Yin J Qian L Shen J : From value co-creation to value co-destruction? The case of dockless bike sharing in China. *Transp. Res. D Trans. Environ.* 2019;71:169–185. 10.1016/j.trd.2018.12.004

