# Supplementary material for: Precursors of interactional value formation through digital banking interactions
Source: F1000Res. 2025 Feb 26;14:235. [Version 1] doi: 10.12688/f1000research.161114.1 (PMC12163366; doi:10.12688/f1000research.161114.1)
Supplement: Peer review history

# Reviewer report: Reviewer response for version 1

**DOI:** 10.5256/f1000research.177106.r387969

**Published:** 2025-06-12

**Authors:** S Anand

**Affiliations:** 1 College of Banking and Financial Studies, Muscat, Oman

The article titled *“Precursors of Interactional Value Formation through Digital Banking Interactions”* presents a well-structured and empirically sound investigation into the key antecedents—relationship quality, communication, and knowledge—affecting value co-creation in digital banking. The authors effectively employ a robust quantitative approach, utilizing a substantial sample size and appropriate statistical techniques (PLS-SEM). The findings are clearly presented and supported by relevant, current literature, with knowledge emerging as the strongest driver of interactional value formation. The study makes a significant contribution to the limited empirical research on digital banking interactions, particularly within the Indian context.

 To further enhance the manuscript, the authors could consider simplifying some of the technical terms and providing brief explanations for key concepts, such as “interactional value formation” and “co-creation,” especially for readers new to the topic. Including a concise summary table of key findings would also enhance clarity and readability. Additionally, the limitations section could be slightly expanded to mention potential response biases associated with self-reported data and suggest how future studies might address this issue.  Expanding practical implications—particularly on how banks can build employee knowledge and relational quality in digital settings—would add value for practitioners. Lastly, the authors may consider adding 2–3 simple suggestions for how banks can improve customer knowledge and trust in digital interactions, such as staff training or improving website content. These small additions will strengthen the practical relevance of the study. Overall, the manuscript is of high quality and merits acceptance with minor revisions.

Is the work clearly and accurately presented and does it cite the current literature?

Yes

If applicable, is the statistical analysis and its interpretation appropriate?

Yes

Are all the source data underlying the results available to ensure full reproducibility?

Yes

Is the study design appropriate and is the work technically sound?

Yes

Are the conclusions drawn adequately supported by the results?

Yes

Are sufficient details of methods and analysis provided to allow replication by others?

Yes

Reviewer Expertise:

Finance, Accounting, Banking, Behavioural Finance, Capital Market, Investments, Service Quality

I confirm that I have read this submission and believe that I have an appropriate level of expertise to confirm that it is of an acceptable scientific standard.

# Reviewer report: Reviewer response for version 1

**DOI:** 10.5256/f1000research.177106.r387968

**Published:** 2025-06-05

**Authors:** Prakash H S

**Affiliations:** 1 SDM College of Engineering and Technology, Dharwad, India

The article is thorough and uses a tried-and-true approach to address the stated goal. The research methodology used in this article is very strong, and the data is analyzed using sophisticated statistical tools. The article's conclusion is highly original and useful. Knowing and comprehending the significance of IVF in real life is highly pertinent.

Is the work clearly and accurately presented and does it cite the current literature?

Yes

If applicable, is the statistical analysis and its interpretation appropriate?

Yes

Are all the source data underlying the results available to ensure full reproducibility?

Yes

Is the study design appropriate and is the work technically sound?

Yes

Are the conclusions drawn adequately supported by the results?

Yes

Are sufficient details of methods and analysis provided to allow replication by others?

Yes

Reviewer Expertise:

Analysis of consumer preference, consumer decision-making process, rural marketing, and service quality analysis.

I confirm that I have read this submission and believe that I have an appropriate level of expertise to confirm that it is of an acceptable scientific standard.

## References

[1] Tauh & Basri (2025).
